# Mutation of *EpCAM* leads to intestinal barrier and ion transport dysfunction

**DOI:** 10.1007/s00109-014-1239-x

**Published:** 2014-12-09

**Authors:** Philip A. Kozan, Matthew D. McGeough, Carla A. Peña, James L. Mueller, Kim E. Barrett, Ronald R. Marchelletta, Mamata Sivagnanam

**Affiliations:** 1 Division of Gastroenterology, Department of Medicine, University of California San Diego, San Diego, CA USA; 2 Division of Allergy, Immunology, and Rheumatology, Department of Pediatrics and Medicine, University of California San Diego, San Diego, CA USA; 3 Ludwig Institute of Cancer Research, San Diego Branch, San Diego, CA USA; 4 Division of Gastroenterology, Hepatology, and Nutrition, Department of Pediatrics, Rady Children’s Hospital, University of California San Diego, La Jolla, San Diego, CA USA

**Keywords:** Congenital tufting enteropathy, EpCAM, Ion transport, Barrier function

## Abstract

**Abstract:**

Congenital tufting enteropathy (CTE) is a devastating diarrheal disease seen in infancy that is typically associated with villous changes and the appearance of epithelial tufts. We previously found mutations in epithelial cell adhesion molecule (*EpCAM*) to be causative in CTE. We developed a knock-down cell model of CTE through transfection of an *EpCAM* shRNA construct into T84 colonic epithelial cells to elucidate the in vitro role of EpCAM in barrier function and ion transport. Cells with EpCAM deficiency exhibited decreased electrical resistance, increased permeability, and decreased ion transport. Based on mutations in CTE patients, an in vivo mouse model was developed, with tamoxifen-inducible deletion of exon 4 in *Epcam* resulting in mutant protein with decreased expression. Tamoxifen treatment of *Epcam* ^Δ4/Δ4^ mice resulted in pathological features of villous atrophy and epithelial tufts, similar to those in human CTE patients, within 4 days post induction. *Epcam* ^Δ4/Δ4^ mice also showed decreased expression of tight junctional proteins, increased permeability, and decreased ion transport in the intestines. Taken together, these findings reveal mechanisms that may underlie disease in CTE.

**Key messages:**

- Knock-down EpCAM cell model of congenital tufting enteropathy was developed.
- In vivo inducible mouse model was developed resulting in mutant EpCAM protein.
- Cells with EpCAM deficiency demonstrated barrier and ion transport dysfunction.
- Tamoxifen-treated *Epcam* ^Δ4/Δ4^ mice demonstrated pathological features.
- *Epcam* ^Δ4/Δ4^ mice showed improper barrier function and ion transport.

**Electronic supplementary material:**

The online version of this article (doi:10.1007/s00109-014-1239-x) contains supplementary material, which is available to authorized users.

## Introduction

Congenital tufting enteropathy (CTE) is a severe intractable diarrheal disease presenting in the neonatal period with chronic watery diarrhea, imbalances in electrolytes, and impaired growth. The prevalence of CTE is thought to be 1/50,000–1/100,000 live births in Western Europe [1]. CTE is an autosomal recessive disease, often seen in families with a history of consanguinity [2]. A diagnosis of CTE is made with recognition of changes in the villi of the small intestinal epithelium. CTE is usually accompanied by villous atrophy, crypt hyperplasia, formation of focal epithelial tufts (bunching of enterocytes) in the small intestinal and colonic mucosae, and absent or mild inflammation [3, 4]. CTE often results in intestinal failure. Patients must rely on total parenteral nutrition to receive necessary caloric intake for growth and development, as no therapies exist at this time [1]. Periods of prolonged parenteral nutrition are not optimal due to both quality of life and health considerations, such as vascular complications and liver disease [5, 6].

Epithelial cell adhesion molecule (EpCAM) is a cell adhesion molecule involved in cellular communication [7]. EpCAM was first recognized as an antigen overexpressed on human carcinoma cells in the digestive tract, breasts, and kidneys [8]. In healthy adult tissue, EpCAM is typically expressed on the basolateral surface of simple and pseudostratified epithelial cells in the gastrointestinal, respiratory tracts, and reproductive systems. EpCAM has been shown to be involved in cellular adhesion and proliferation, but its exact role in intestinal function has not been fully elucidated [9, 10].

Sivagnanam et al. first identified mutations in *EpCAM* as causative in CTE, and this has been confirmed by other groups [11–14]. CTE patients express mutant EpCAM at significantly decreased levels in biopsied intestinal tissue as seen by immunohistochemistry and Western blot [11]. Staining with antibodies to EpCAM has been established as a diagnostic tool in CTE [4, 15].

To maintain intestinal homeostasis, normal barrier function is essential. The barrier formed between epithelial cells is partly comprised of the apically located tight junction complex. Tight junctional proteins include occludin, claudins, junctional adhesion molecules (JAMs), and zonula occludens (ZO) proteins [9]. ZO proteins link the membrane proteins, occludin and claudins, to the actin cytoskeleton. In the absence of ZO’s, cells fail to form effective tight junctions [16, 17]. Occludin is important in regulating the paracellular barrier via binding of its C-terminus to ZO-1, allowing localization of occludin to the tight junctions [18, 19]. EpCAM has also previously been shown to be involved in the formation of tight junctions through the recruitment and regulation of claudin proteins [9, 15, 20]. In this study, we investigated how disruption of EpCAM affects tight junctions and barrier function, as they relate to the observed phenotype of CTE.

The T84 colonic adenocarcinoma cell line has been widely used as a model for studies of epithelial electrolyte transport and barrier function. When T84 cells are grown as a monolayer, they form tight junctions and display secretagogue-sensitive chloride secretion as a model for secretory cells within the colonic crypt [21]. In this study, we generated an shRNA transfected T84 cell line in which *EpCAM* was stably knocked-down (KD) to elucidate the role of *EpCAM* in tight junction formation and secretory function. As EpCAM is expressed in both the small and large intestines [4], knock-down of EpCAM in colonic T84 cells is an appropriate in vitro model to investigate the mechanism of intestinal dysfunction in the emergence of diarrhea with EpCAM deficiency.

EpCAM is highly conserved in mammals, and its distribution in mice is similar to that of human EpCAM with highest expression in the intestines [22]. We generated an in vivo inducible mouse model of CTE with a mutation that corresponds to a common *EpCAM* mutation found in patients, in which a homozygous G>A substitution at the donor splice site of exon 4 results in a mRNA splice product lacking exon 4 [15, 23]. Unlike our previous model [15], inducible deletion of exon 4 allows for extended survival, permitting us to further elucidate the in vivo role of EpCAM in barrier formation and electrolyte transport. With this mouse model, we sought to validate the T84 cell model in studying the mechanisms of CTE and to further characterize the intestinal effects of mutant EpCAM from a post developmental standpoint. We hypothesized that both the KD cells and mutant murine model would show attenuated expression of tight junctional proteins, altered ion transport, and increased permeability of the intestinal barrier.

## Methods

### Development of inducible epcam^Δ4/Δ4^ mice

The *Epcam* targeting construct was developed as previously described [15]. Mice homozygous for the mutant *Epcam* Δ4 construct, in which the neomycin-resistant positive selection marker had been previously removed via Flip recombinase, were bred to B6.Cg-Tg(Cre/Esr1)5Amc/J mice obtained from Jackson Labs, Bar Harbor, ME. This allowed for Cre-LoxP recombination and efficient deletion of exon 4 in *Epcam* following administration of tamoxifen [24]. Mice were orally gavaged with 25 mg/kg tamoxifen free base (MP Biomedicals, Solon, OH) in 90 % sunflower seed oil from *Helianthus annus* (Sigma, St. Louis, MO) /10 % ethanol once daily for two consecutive days, generating *Epcam* ^Δ4/Δ4^ mice. *Epcam* ^WT/WT^ mice orally gavaged with tamoxifen (as above) served as controls for all experiments (WT). All studies were approved by the UCSD Institutional Animal Care and Use Committee.

### Cell culture

T84 cells were cultured in 1:1 Dulbecco’s modified Eagle’s Medium/F-12 Ham’s medium with 15 mM l-glutamine (Mediatech Inc., Manassas, VA), 5 % bovine calf serum (Invitrogen, Grand Island, NY), 1 % penicillin-streptomycin (Mediatech Inc.) and maintained according to a standard protocol [25].

Specific KD of *Epcam* in cells was accomplished with SureSilencing shRNA plasmids (SABiosciences Corporation, Frederick, MD). High efficiency KD was achieved via Amaxa electroporation. Two microgram of shRNA plasmid was added to T84 cells, and electroporation was performed according to manufacturer recommendations. Cells were grown in selective media (500 μg/ml G-418 sulfate, Mediatech Inc.) for 3 weeks to generate stable transfectants. In order to retain the KD phenotype in cells transfected with shRNA, 40 μg/mL of G418 sulfate was retained in the medium thereafter.

For resistance and permeability studies, cells were grown on semipermeable 12-mm Millicell-HA culture plate inserts with 0.5 × 10^6^ cells added per insert. Cells were maintained in media as above. Cell culture media was changed every 3 days for approximately 2 weeks until cells formed a monolayer [25].

### Electrical resistance

To assess monolayer integrity, a voltohmmeter (World Precision Instruments, Sarasota, FL) was used to measure transepithelial electrical resistance (TER) across the monolayer. The monolayer was considered mature when the TER of control T84 cells reached a peak value (600 ohms.cm^2^). TERs were recorded for control and KD cell monolayers every day until 2 days past maturity, which was at about 2 weeks.

Four days after initial tamoxifen induction, *Epcam* ^*Δ4/Δ4*^ and control mice were sacrificed by cervical dislocation and full-thickness segments of distal ileum and proximal colon were mounted in Ussing chambers (Physiological Instruments, San Diego, CA). A transepithelial current pulse of 10 μA was administered to assess tissue viability, and TER was calculated using Ohm’s law.

### Permeability

When a mature monolayer was confirmed by TER measurement of controls, macromolecular permeability was assessed in control and KD T84 cell monolayers. Fluorescein isothiocyanate (FITC)-dextran 4kD (Sigma) was added to the apical medium bathing semipermeable inserts at a final concentration of 1 mg/mL. At each hour thereafter, for 3 h, the basolateral medium was sampled. For tissue studies, FITC-dextran was added to the serosal side (1 mg/mL) of tissue segments mounted in Ussing chambers and the mucosal medium sampled at hourly intervals thereafter. All samples were analyzed for FITC levels with a Spectramax M2 Absorbance Microplate reader (Molecular Devices, Sunnyvale, CA) calibrated with appropriate standards.

### Ion transport

To assess ion transport responses, T84 cells grown on semipermeable membranes and distal ileum and proximal colon tissues from *Epcam* ^*Δ4/Δ4*^ mice and control animals were mounted into Ussing chambers according to protocol [26] and bubbled with 95, 5 % CO_2_. Once mounted in the chambers (cell window area: 0.6 cm^2^, mouse tissue window area: 0.09 cm^2^), inserts/tissue were allowed 20 min of equilibration. Changes in short circuit current (ΔIsc) were measured after sequential exposure to forskolin (fsk) and carbachol (cch), which are cAMP- and calcium-dependent chloride secretagogues, respectively.

### Western blotting

T84 cell monolayers were suspended in ice-cold lysis buffer (50 mM Tris, 150 mM NaCl, 0.1 % SDS, 0.5 % sodium deoxycholate, 20 μM NaF, 1 mM EDTA, 1 μg/ml antipain, 1 μg/ml pepstatin, 1 μg/ml leupeptin, 1 mM NaVO_3_, and 100 μg/ml phenylmethylsulfonyl fluoride), vortexed thoroughly, and lysed using a 22-gauge needle. The lysates were centrifuged at 10,000 rpm for 10 min to remove insoluble material. An aliquot was removed from each sample to determine protein concentration using the Bio-Rad (Hercules, CA) protein assay, according to the instructions of the manufacturer, and analyzed by Spectramax M2 Absorbance Microplate reader. Samples were resuspended in loading buffer (50 mM Tris -pH 6.8, 2 % SDS, 100 mM dithiothreitol, 0.2 % bromphenol blue, and 20 % glycerol).

Distal ileum and proximal colon were placed in nonyl phenoxypolyethoxyethanol-40(NP-40) buffer containing 0.9 % NaCl, 10 % glycerol, 50 mM Tris pH2.8, 0.1 % NP40, 5 mM EDTA, 20 μM NaF, 1 μg/ml antipain, 1 μg/ml pepstatin, 1 μg/ml leupeptin, 1 mM NaVO_3_, and 100 μg/ml phenylmethylsulfonyl fluoride and lysed using a mini bead beater (BioSpec Products, Bartlesville, OK). The lysate was centrifuged and the supernatant containing the protein was removed, with protein concentration determined as above. Samples were resuspended in loading buffer.

Cell and tissue lysates were diluted with a 5 to 1 ratio of lysate to loading buffer and loaded onto Mini-Protean TGX precast gels (BioRad), electrophoresed, then transferred onto a nitrocellulose membrane. Membranes were blocked with 5 % bovine serum albumin/TBST for studies of occludin, EpCAM, and ZO-1, and 5 % dry milk/TBST for studies of the Na/K/2Cl cotransporter, NKCC-1 and the cystic fibrosis transmembrane conductance regulator, CFTR.

Western blotting was performed using mouse antibodies to occludin (Invitrogen), goat antibodies to NKCC-1 (Santa Cruz Biotechnology, Dallas, TX), rabbit antibodies to ZO-1 (Invitrogen), CFTR (GenTex, Zeeland, MI), and EpCAM (abcam, Cambridge, MA) diluted at 1:1000. A mouse monoclonal antibody to β-actin at 1:2000 (Sigma) was used to correct for loading. Horseradish peroxidase-conjugated anti-mouse, anti-rabbit, and anti-goat IgG (Cell Signaling Technologies, Beverly, MA) secondary antibodies were used at 1:2000 dilutions. A semiquantitative measurement of band density was performed using Scion Image for Windows software.

### Q-PCR

Total RNA from T84 cells was isolated using RNeasy Mini kits (Qiagen, Valencia). Distal ileum and proximal colon tissues were excised and homogenized in TRIzol (Invitrogen) with a bead beater. RNA was extracted with chloroform, followed by 70 % ethanol and an RNeasy Mini kit (Qiagen). First strand cDNA was synthesized with iScript cDNA Synthesis kit (Bio Rad) using the recommended protocol. Real-time PCR reactions were set up using FastStart Universal SYBR Green Master Mix (Invitrogen) and thermal cycling performed on a StepOnePlus Real-Time PCR System using Step One software v2.0 (Applied Biosystems, Carlsbad, CA). Primers were designed and obtained from IDT (Integrated DNA Technologies, Coralville, IA, Supp. Table 1). All primers were diluted to a concentration of 100 μM. Glyceraldehyde 3-phosphate dehydrogenase (GAPDH) and villin were used as endogenous controls.

### Immunofluorescence

Control and EpCAM KD T84 cells were grown on glass coverslips sterilized with 70 % ethanol and a Bunsen burner flame. Cells were washed with PBS and fixed with 3.7 % paraformaldehyde for 30 min at room temperature. Tissue sections were blocked using 5 % bovine serum albumin/PBS and then incubated overnight at 4 °C with antibodies to actin (Sigma), NKCC-1 (Santa Cruz Biotechnology), or EpCAM (abcam) diluted at 1:200. Secondary detection was performed by incubation with Alexa Fluor 568-conjugated donkey anti-mouse or Alexa Fluor 568-conjugated goat anti-rabbit antibodies, Alexa Fluor 647 donkey anti-goat all diluted at 1:200, and Hoechst 33258 (Invitrogen).

Distal ileum and proximal colon were excised and fixed with 4 % paraformaldehyde for 24 h at room temperature, then paraffin-embedded and sectioned onto glass slides. De-paraffination was performed and samples were heated in 10 mM sodium citrate buffer. Blocking was performed as previously described [27]. Tissue sections were incubated with antibodies (1:200) to NKCC1 (Santa Cruz Biotechnology) or actin (Sigma) for 1 h. Secondary detection was performed using secondary, Alexa Fluor 568-conjugated donkey anti-mouse (1:200) or Alexa Fluor 488-conjugated donkey anti-rabbit (1:200) antibodies and Hoechst 33258 (Invitrogen). Imaging was performed using a Zeiss LSM 510 confocal imaging system, 25× Plan-Apo, 0.8 Numerical aperture. The imaging medium used was ProLong gold anti-fade reagent (Invitrogen), and imaging was done at room temperature using Zeiss LSM Image Acquisition software.

### Statistical analysis

Linear regression, one-way Anova, and *t* test analyses were performed using GraphPad Prism version 4.00 for Windows (GraphPad Software, La Jolla, CA).

## Results

### Knock-down of EpCAM in T84 cells

**Fig. 1:**
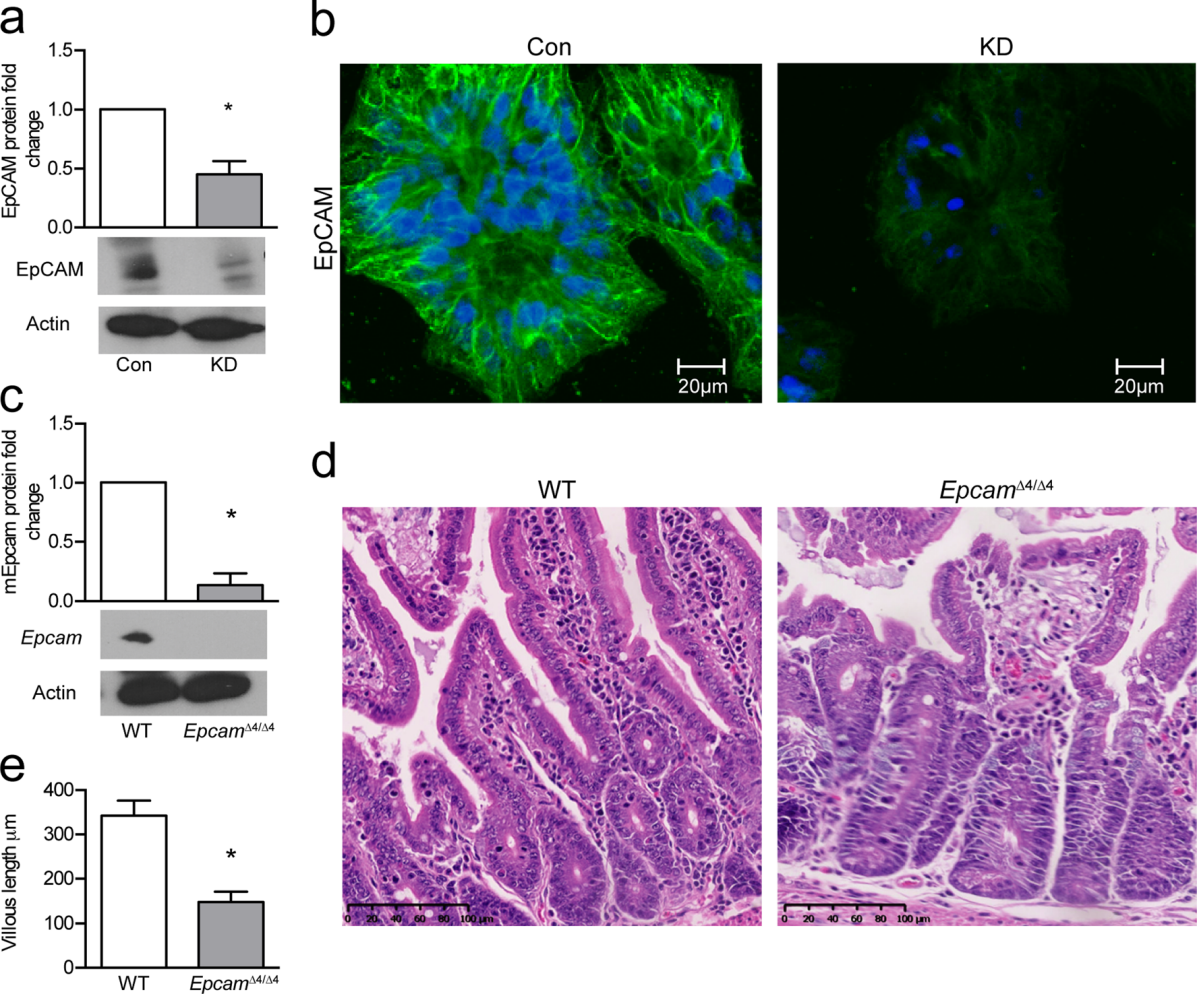
Targeting EpCAM in cell and murine models results in reduced EpCAM expression and morphological changes in murine intestine. **a** Quantification and Western blot of EpCAM in control and KD-EpCAM T84 cells (*n* = 3). **b** Fluorescent immunohistochemistry reveals a decrease in EpCAM fluorescence in KD-EpCAM T84 cells. EpCAM *green*, DAPI *blue* (*n* = 3). **c** Quantification and Western blot of EpCAM in inducible murine model (*n* = 3). **d** H&E staining of ileum shows morphological changes in villi and crypts of *Epcam* ^*Δ4/Δ4*^ mice. **e** *Epcam* ^*Δ4/Δ4*^ mice exhibit decreased villous length (*n* = 6). Fold changes were compared using Student’s paired *t* test. *p < 0.05, *bars* indicate standard error of the mean (SEM)

To determine the role of EpCAM in barrier function, we knocked-down this protein in T84 cells using shRNA that targeted EpCAM. KD cells showed decreased levels of EpCAM by Western blot compared to control cells (Con) treated with scrambled shRNA (Fig. 1a).

Confocal microscopy was used to compare the expression and localization of EpCAM between KD and control T84 cells. As expected, EpCAM fluorescence was reduced in KD cells (Fig. 1b), but the residual EpCAM in KD cells still appeared to localize to intercellular contacts, as seen in control cells.

### Mutation of Epcam in mice results in intestinal defects

Following oral gavage with tamoxifen, small intestinal tissue from *Epcam* ^Δ4/Δ4^ mice was processed for Western blotting, revealing a significant decrease in EpCAM expression compared to controls (Fig. 1c). Small intestinal tissue from *Epcam* ^Δ4/Δ4^ and control mice was evaluated histologically. Tissue from *Epcam* ^Δ4/Δ4^ mice displayed several characteristics similar to those seen in CTE patients and in our previous constitutive mouse model [15], including villous atrophy and bunching of enterocytes leading to the formation of tufts on villi (Fig. 1d). Quantitatively, there was a significant decrease in villous length in *Epcam* ^Δ4/Δ4^ mice (Fig. 1e).

### Knock-down of EpCAM results in barrier dysfunction

**Fig. 2:**
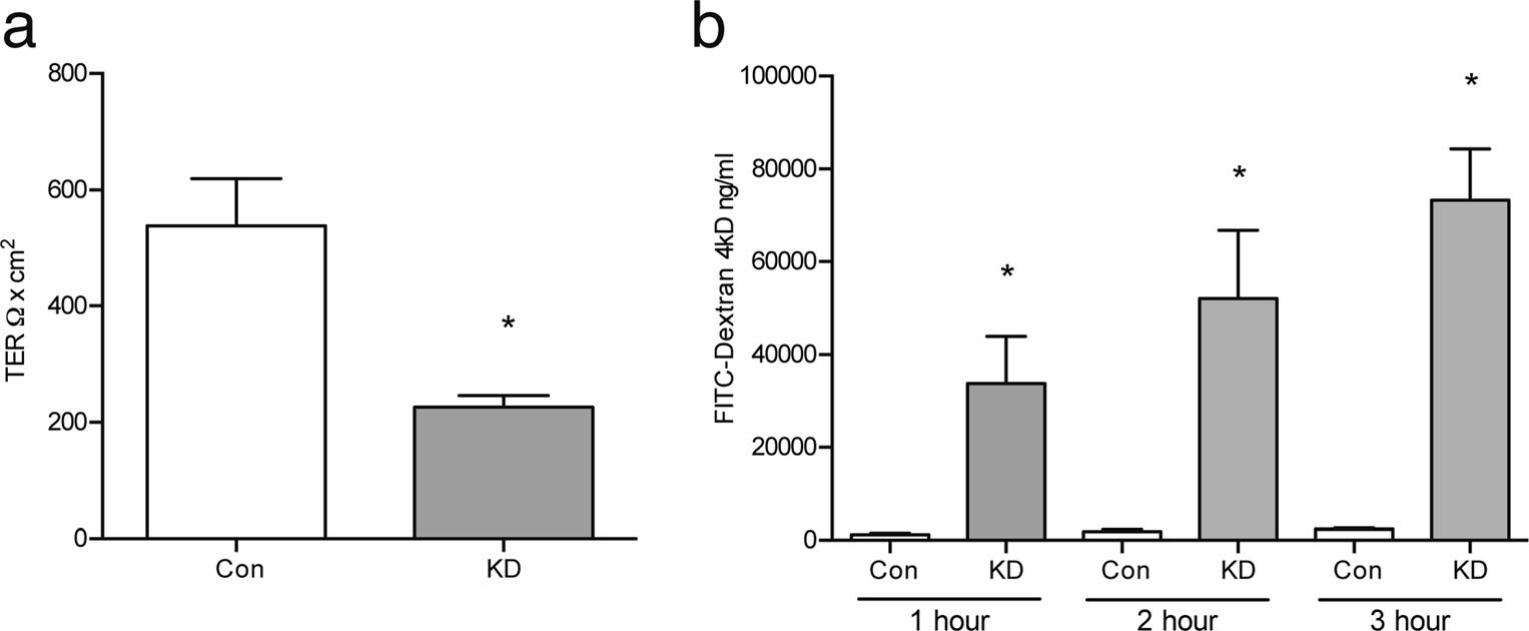
Knockdown of EpCAM attenuates TER and permeability. **a** TER of T84 cells was decreased in KD-EpCAM cells (*p < 0.05, *n* = 8). **b** FITC-4kD permeability was significantly increased in KD-EpCAM cells (*p < 0.0001, student’s paired *t* test, *n* = 6). *Bars* indicate SEM

KD and control T84 monolayers were evaluated for TER, permeability, and expression of tight junctional proteins. TER was significantly decreased in KD cells (Fig. 2a) compared to control cells receiving scrambled shRNA. The two cell populations were also examined for macromolecular permeability using FITC-dextran. At each time point examined, KD cells showed significant increases in FITC-dextran permeability compared to controls (Fig. 2b).

**Fig. 3:**
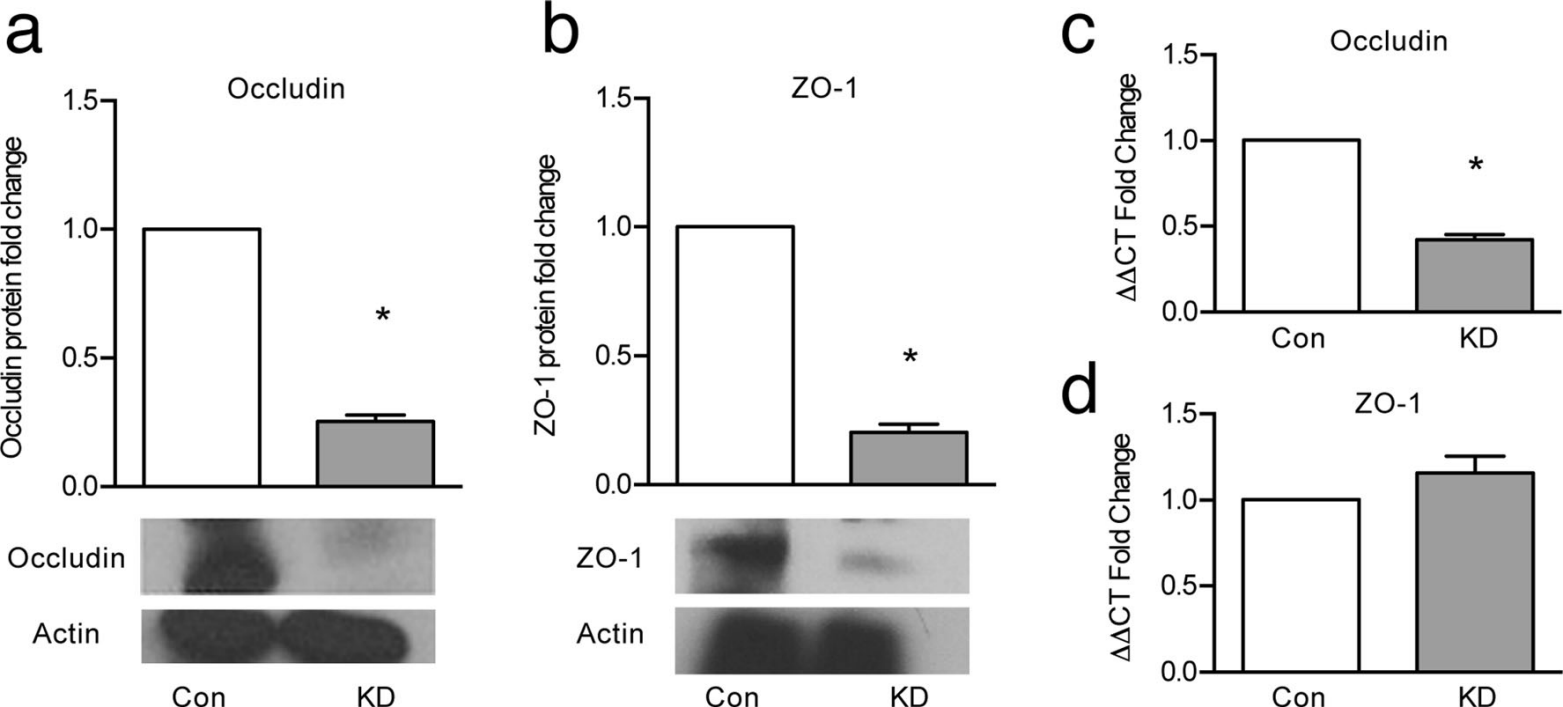
Tight junction proteins are decreased in KD-EpCAM cell lines. **a** Quantification and Western blot of occludin in control and KD-EpCAM cells (*n* = 3). **b** Quantification and Western blot of ZO-1 in control and KD-EpCAM cells (*n* = 3). **c** Occludin mRNA levels by qPCR in KD-EpCAM cells compared with control T84 cells (*n* = 3). **d** ZO-1 RNA levels by qPCR in KD-EpCAM cells compared with control T84 cells (*n* = 3). Fold changes were compared using Student’s paired *t* test. *p < 0.05, *bars* indicate SEM

To investigate possible mechanisms that might explain the decrement in barrier function when EpCAM was decreased, levels of occludin and ZO-1 were examined. Expression of both proteins was decreased in KD compared to control cells (Fig.3a, b). At the mRNA level, expression of occludin was significantly decreased in KD cells (Fig. 3c), but there was no significant effect of EpCAM KD on mRNA levels for ZO-1 (Fig. 3d).

**Fig. 4:**
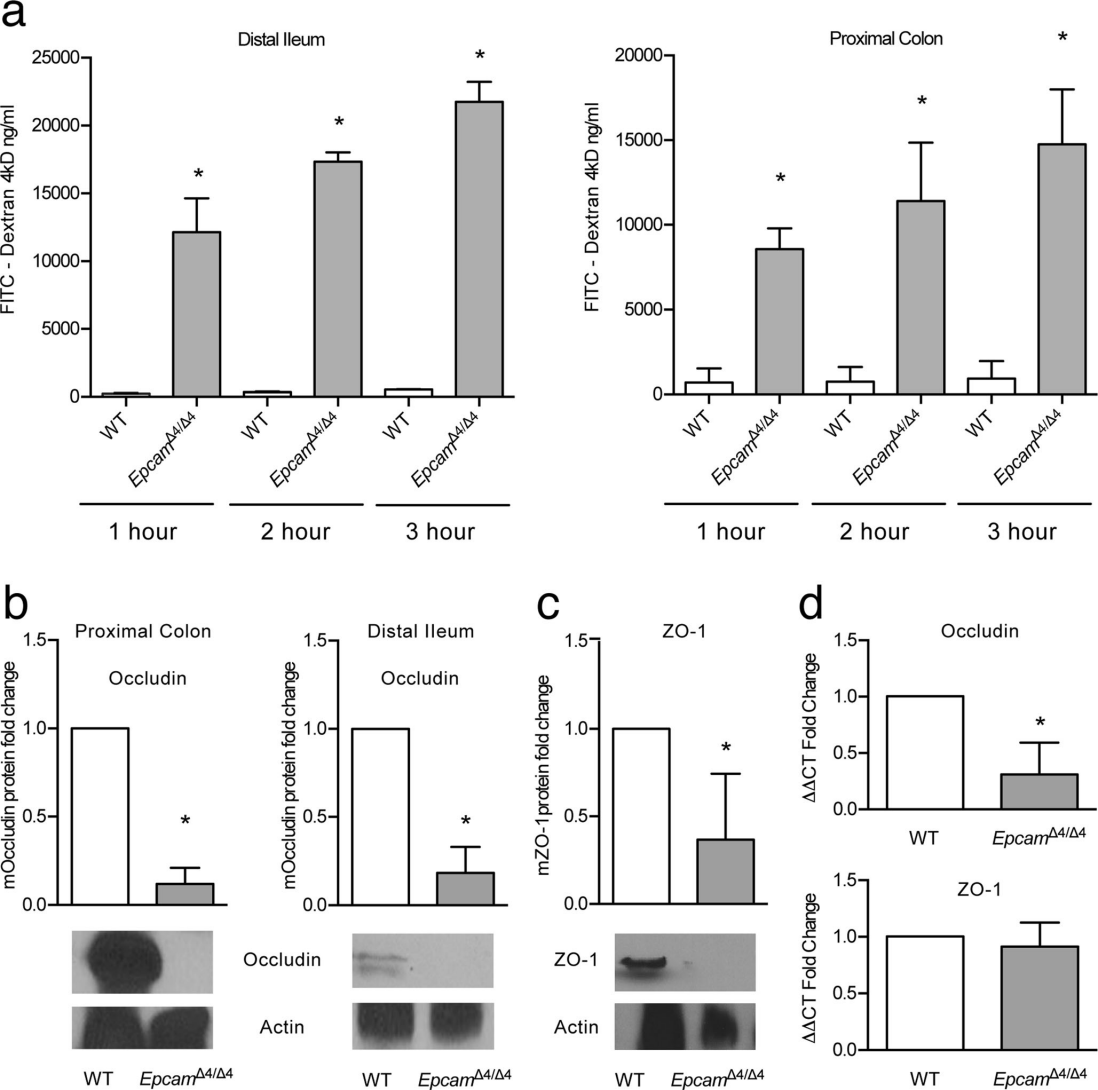
Mutated *Epcam* in mice has deleterious effects on permeability and decreases tight junction proteins. **a** Permeability of FITC-4kD is significantly increased in both distal ileum and proximal colon (*p < 0.0001, *n* = 3). **b** Occludin levels in the proximal colon and distal ileum by Western blot and quantification of fold change (*p < 0.05, *n* = 3). **c** ZO-1 levels in the distal ileum by Western blot and quantification of fold change (*p < 0.05, *n* = 4). **d** Occludin and ZO-1 mRNA levels by qPCR in the small intestines of *Epcam* ^*Δ4/Δ4*^ mice (*p < 0.05, *n* = 4). Fold changes were compared using Student’s paired *t* test. *Bars* indicate SEM

To determine to what extent the functional effects of EpCAM KD in T84 cells were also seen in vivo, we studied barrier function in intestinal segments obtained from tamoxifen-treated *Epcam* ^Δ4/Δ4^ mice and control animals. Permeability to FITC-dextran was significantly higher in both distal ileum and proximal colon from *Epcam* ^Δ4/Δ4^ mice than in control animals (Fig. 4a).

To corroborate these findings, the expression of tight junctional proteins was measured in the murine model. Western blot revealed that expression of occludin and ZO-1 was decreased in the small intestine of *Epcam* ^Δ4/Δ4^ mice compared with controls (Fig. 4b, c). On the other hand, evaluation of mRNA for ZO-1 by qPCR showed comparable expression levels in *Epcam* ^Δ4/Δ4^ and control animals while there was decreased expression of mRNA for occludin (Fig. 4d), consistent with findings in the in vitro model described above.

### Knock-down of EpCAM results in ion transport dysfunction

**Fig. 5:**
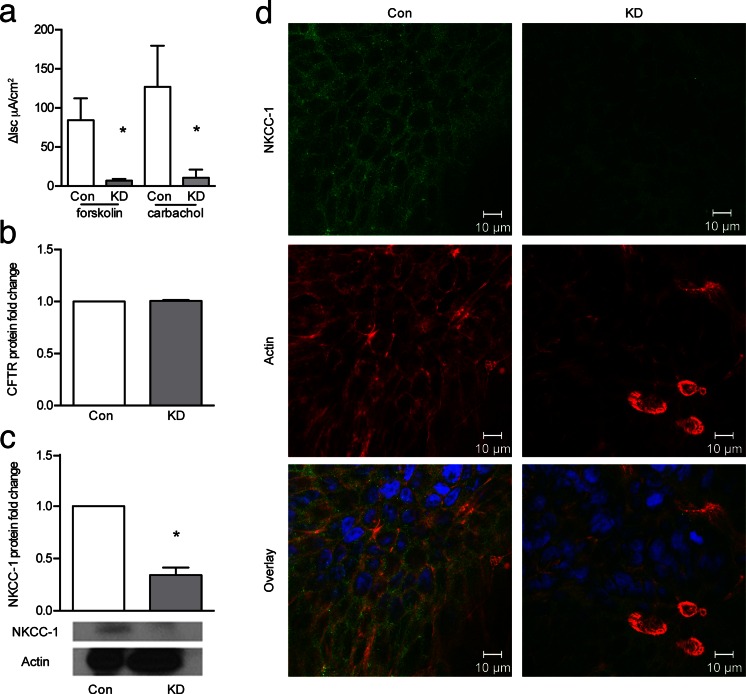
Knockdown of EpCAM results in decreased ion transport. **a** ΔIsc in response to forskolin (fsk) and carbachol (cch) show decreased responses in KD-EpCAM cells (*p < 0.0001, *n* = 6). **b** CFTR measured by Western blot and quantified for fold change (*n* = 3). **c** NKCC-1 measured by Western blot and quantified for fold change (*p < 0.05, *n* = 3). **d** Fluorescent immunohistochemistry of NKCC-1 showed normal localization but decreased fluorescence in KD-EpCAM cells. NKCC-1 *green*, actin *red*, DAPI *blue* (*n* = 4). Data were compared using Student’s paired *t* test. *Bars* indicate SEM

To explore potential mechanisms for the severe diarrhea in CTE, we studied chloride secretion in T84 KD cells compared with controls. Cell monolayers were mounted in Ussing chambers and stimulated with fsk and cch (Fig. 5a). Changes in short circuit current (ΔIsc), reflective of net chloride secretion in response to these agonists, were measured. Surprisingly, chloride secretion evoked by either fsk or cch was reduced in cells with reduced EpCAM expression compared to control cells (Fig. 5a).

To investigate a possible basis for the reduction in chloride secretion, the expression of key transporters, CFTR and NKCC-1, was studied by Western blotting. There was no appreciable difference in the expression of CFTR between control and KD cells (Fig. 5b). In contrast, levels of NKCC-1 were significantly decreased in KD cells compared with controls (Fig. 5c). Further investigation of NKCC-1 was performed with confocal microscopy. A decrease in fluorescence for NKCC-1 was observed in KD cells, although the transporter apparently remained confined to the basolateral domain as seen in control cells (Fig. 5d). Interestingly, we also found that the actin in KD cells was disorganized and aggregated into clumps vs. the typical cobble stone pattern of actin localization seen in control cells (Fig. 5d).

**Fig. 6:**
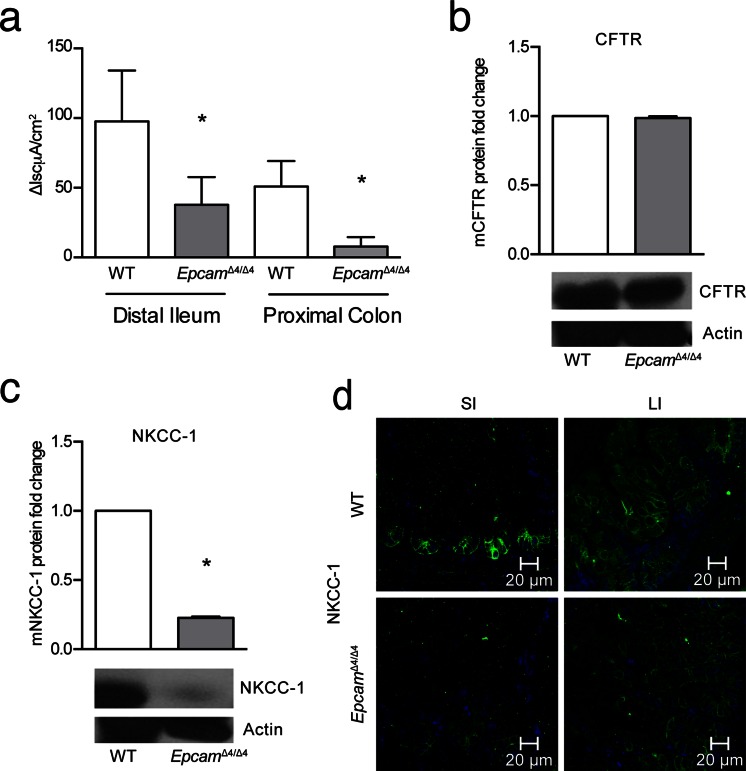
Mutated EpCAM causes ion transport dysfunction in the murine model. **a** ΔIsc in response to forskolin in the distal ileum and proximal colon was decreased in mice with mutant EpCAM (*n* = 5). **b** CFTR in the small intestine measured by Western blot and quantified for fold change (*n* = 3). **c** Protein expression and quantification of NKCC-1 in the small intestines (*n* = 3). **d** Immunofluorescence of NKCC-1 in the small and large intestines shows decreased fluorescence in mice with mutated EpCAM (*n* = 3). NKCC-1 *green*, DAPI *blue*, *SI* small intestine, *LI* large intestine. Data were compared using Student’s paired *t* test. *p < 0.05, *bars* indicate SEM

Investigation of ion transport in tissues from *Epcam* ^Δ4/Δ4^ mice revealed similar findings. Sections of distal ileum and proximal colon were mounted in Ussing chambers and stimulated with fsk. Although both absorptive and secretory transport processes are present in these tissues, chloride secretion should be the predominant electrogenic process in these segments, particularly in response to fsk, and so, ΔIsc can be taken as a proxy for this transport event. In both segments, the chloride secretory response to fsk was reduced in tissues from *Epcam* ^Δ4/Δ4^ mice following tamoxifen treatment compared to control animals (Fig. 6a).

CFTR and NKCC-1 were analyzed by Western blot in these tissues, showing no significant difference in the expression of CFTR (Fig. 6b), but significantly decreased expression of NKCC-1 within the distal ileum (Fig. 6c). Immunohistochemistry revealed decreased fluorescence for NKCC-1 throughout the small and large intestines in *Epcam* ^Δ4/Δ4^ mice compared to controls, although residual NKCC-1 remained localized to the basolateral membrane as seen in control mice (Fig. 6d).

## Discussion

Congenital tufting enteropathy, a disorder caused by mutations in *EpCAM*, results in a serious clinical phenotype that is debilitating to children. Through the use of in vitro and in vivo models of CTE, we have demonstrated that mutations in *EpCAM* result in its reduced expression, also leading to intestinal epithelial dysfunction encompassing compromised resistance, macromolecular permeability, tight junction integrity, and changes in ion transport.

Although the T84 cell line cannot model absorptive transport in the intestine, these cells form a polarized monolayer with tight junctions and display robust chloride secretory responses to both cAMP- and calcium-dependent agonists [28, 29]. KD of EpCAM in T84 cells significantly impaired their ability to develop normal barrier properties. Similarly, in our inducible mouse model, *Epcam* ^*Δ4/Δ4*^ mice exhibited a leaky epithelial barrier. In light of these results and previous knowledge that claudin proteins are downregulated in the presence of mutant EpCAM [15, 20], we evaluated expression of key tight junction proteins to explore the mechanism of barrier dysfunction. ZO-1, a protein essential for the assembly of tight junctions in epithelial cells [16, 30] is known to interact with the C-terminus of occludin [31]. Occludin is involved in tissue resistance as evidenced by previous studies showing occludin-dependent increases in TER [32]. Protein levels for both occludin and ZO-1 were decreased when EpCAM levels were reduced in both our cell and mouse models. For occludin, a concomitant decrease in mRNA suggests *EpCAM* may stimulate its transcription through a direct or indirect mechanism. On the other hand, a post-transcriptional impact of EpCAM on ZO-1 must presumably occur based on the unchanged RNA levels. This warrants future studies into the protein-protein interactions of EpCAM, as it has been previously shown to control the composition and function of tight junctions [33].

We also studied whether a relative deficit in EpCAM expression would impact ion transport, hypothesizing that an increase in chloride secretion might explain diarrheal symptoms seen in CTE. Changes in ion transport were studied using agonists of chloride secretion: fsk, an activator of the cAMP-dependent pathway causing secretion through CFTR [28], and cch, an activator of a Ca^2+^ dependent pathway that opens a basolateral K^+^ channel, but also results in chloride secretion [29]. However, contrary to our hypothesis, a reduction in EpCAM was accompanied by a reduction in chloride secretion in both T84 cells and *Epcam* ^*Δ4/Δ4*^ mice. This was apparently secondary to a reduction in NKCC-1, but not CFTR, expression. A decrease in the Cl^−^ loading ability of epithelial cells resulting from the decrease in NKCC-1 may explain the decreases in ion transport [34]. Although our findings cannot explain diarrheal symptoms in CTE, they are consistent with reductions in both cAMP- and calcium-dependent chloride secretion that have been reported in various models of colitis [35, 36]. This reduction in chloride secretion has been speculated to contribute to defects in host defense by reducing the normal flushing of crypt contents, thereby protecting the vulnerable stem cell niche [37]. The precise mechanisms whereby a reduction of EpCAM reduces NKCC-1 expression remain to be identified. It will also be of interest to study the impact of decreased EpCAM on the expression of absorptive transporters in the murine model as a possible explanation for fluid loss, as has been reported in some models of infectious diarrhea [38, 39].

While examining NKCC-1 localization in EpCAM KD T84 cells using immunohistochemistry, we found a severe disorganization of the actin cytoskeleton. ZO-1 is important in linking tight junction proteins to the actin cytoskeleton and is also known to associate with α-catenin [17, 40]. Recently, disruption of the ZO-1/α-catenin complex was similarly associated with actin disorganization [41]. It is possible that EpCAM regulates assembly of the ZO-1/α-catenin complex, which could account for the disorganization observed when EpCAM is deficient. Additionally, EpCAM is also known to interact with the actin cytoskeleton directly via α-actinin [42].

Our study in cell and murine models has elucidated an important role for EpCAM in maintaining proper intestinal function. In the case of mutated or absent EpCAM, not only is it not possible for a proper barrier to be maintained, but there is also dysregulation of ion transport. EpCAM is expressed in other organ systems of the body, such as the respiratory tract and kidney, yet these organs do not display obvious dysfunction in CTE patients or our murine model [8]. Although we found decreases in important tight junctional proteins in the small bowel and colon, it may be that EpCAM participates in tissue-specific regulatory pathways or that EpCAM itself is regulated by another pathway outside of the gut that permits normal function of extraintestinal tight junctions even in the absence of EpCAM. Additionally, the intestine-specific pathology may be explained by EpCAM’s differential expression levels throughout the body, with areas such as the small intestines and colon having the highest expression [43]. Previous studies have shown increased levels of occludin and ZO-1 in lung tissue compared to the colon and small bowel [44]. Thus, some tissues in which EpCAM is normally expressed may have tight junctions that are more resistant to the loss of a regulatory molecule such as EpCAM.

Our study nevertheless builds on previous understanding of the function of EpCAM in the intestine to demonstrate that EpCAM likely plays a critical role in the formation of tight junctions and the regulation of specific ion transporters in intestinal epithelial cells. Moreover, similar findings in the cell and murine models suggest that the cell model of CTE can be deployed for efficient mechanistic studies of CTE. Our findings are also suggestive that, in CTE, the inability to form a robust barrier outweighs secretory problems, possibly explaining the diarrheal phenotype. Future studies examining the role of EpCAM in tight junction formation and how it associates with other cell adhesion molecules should be undertaken. Our findings of actin reorganization likewise warrant further investigation of EpCAM’s role in regulating the cytoskeleton. We aim to achieve a better understanding of how EpCAM disrupts the intestinal barrier in order to develop therapeutic strategies for CTE.

## Electronic supplementary material

Below is the link to the electronic supplementary material.

ESM 1 (PDF 57 kb)
