# Supplementary material for: Mutation of EpCAM leads to intestinal barrier and ion transport dysfunction
Source: J Mol Med (Berl). 2014 Dec 9;93(5):535–45. doi: 10.1007/s00109-014-1239-x (PMC4408367; doi:10.1007/s00109-014-1239-x)
Supplement: Supplementary file 1 — (PDF 57 kb) [file 109_2014_1239_MOESM1_ESM.pdf]

J Mol Med 2015

Mutation of *EpCAM* leads to intestinal barrier and ion transport dysfunction

Authors: *Philip A. Kozan, *Matthew D. McGeough, Carla A. Peña, James L. Mueller, Kim E. Barrett, Ronald R. Marchelletta, Mamata Sivagnanam

*shared first authorship

Corresponding author: Mamata Sivagnanam; Division of Gastroenterology, Hepatology, and Nutrition, Department of Pediatrics, University of California San Diego, La Jolla; Rady Children's Hospital, San Diego; mengineer@ucsd.edu; 858-966-8907

Table 1. Primers

Function	Name of Primer	Primer sequence
Mouse ZO1 RTPCR F	mZO1 forward	CATCTCCAGTCCCTTACCTTTC
Mouse ZO1 RTPCR R	mZO1 reverse	CCTCCAGGCTGACATTAGTTAC
Mouse Villin RTPCR F	mVillin forward	AGCTGCCATCTACACCACACAGAT
Mouse Villin RTPCR R	mVillin reverse	AGTCGCTGGACATCACAGGAGTTT
Human ZO1 RTPCR F	hZO1 forward	CCTGAGTTTGACAGTGGAGTT
Human ZO1 RTPCR R	hZO1 reverse	GCTGAAGGACTCACAGGAATAG
Human Occludin RTPCR F	hOcc forward	GGTTCACTTCTCCCAGTCTTTC
Human Occludin RTPCR R	hOcc reverse	AGACACAATCAACAGGGTTAGG
Human GAPDH RTPCR F	hGAPDH forward	CATGTTCGTCATGGGTGTGAACCA
Human GAPDH RTPCR R	hGAPDH reverse	AGTGATGGCATGGACTGTGGTCAT
